# Identification and validation of a hypoxia- and immune-related prognostic signature for pancreatic cancer

**DOI:** 10.1016/j.bbrep.2025.102205

**Published:** 2025-08-11

**Authors:** Ganghua Yang, Jiawei Yu, Xuqi Li, Fandi Meng, Yong Wan, Zhengyang Lu, Zheng Wang, Qinhong Xu

**Affiliations:** a Department of Geriatric Surgery, The First Affiliated Hospital of Xi'an Jiaotong University, Xi'an, 710061, Shaanxi Province, China; b Department of Hepatobiliary Surgery, The First Affiliated Hospital of Xi'an Jiaotong University, Xi'an, 710061, Shaanxi Province, China; c Department of General Surgery, The First Affiliated Hospital of Xi'an Jiaotong University, Xi'an, 710061, Shaanxi Province, China

**Keywords:** Pancreatic cancer, Prognosis, Hypoxia, Immune, Nomogram

## Abstract

Pancreatic cancer is a lethal disease with a poor prognosis. Immunity and hypoxia are critical characteristics of the tumor microenvironment and are closely associated with cancer prognosis. The present study aimed to identify a novel hypoxia- and immune-related gene signature for the prediction of prognosis in patients with pancreatic cancer. The status of hypoxia or immunity was determined via the NMF method by using data from the TCGA database. A total of 27 hypoxia- or immune-related DEGs were identified. A 6-gene hypoxia- and immune-related prognostic signature (*GALR2*, *AGT*, *MAPT*, *PRKCG*, *CAMK2B* and *PKP1*) was further identified via LASSO regression. Survival analysis revealed that the overall survival of patients with pancreatic cancer was inversely associated with the prognostic signature. A ROC curve indicated the excellent performance of the prognostic signature, with an AUC of 0.713. A similar prognostic value of the prognostic signature was further confirmed in 2 independent GEO cohorts. In addition, a nomogram was constructed with the prognostic signature and clinical factors, including sex, age and histological grade, and the performance of the nomogram was assessed via calibration plots and decision curve analysis. Thus, our study identified a hypoxia- and immune-related prognostic signature and established a nomogram, which may be helpful for survival prediction in patients with pancreatic cancer.

## Highlights

• We established a 6-gene hypoxia- and immune-related prognostic signature that was an independent prognostic factor for pancreatic cancer.
• Survival analysis demonstrated that the prognostic signature was highly valuable for patients with pancreatic cancer.
• A nomogram was constructed with the prognostic signature and clinical characteristics.
• The results of the study provide a promising prognostic signature that may be helpful in practical applications because of the reduced necessity for whole-genome sequencing in patients with pancreatic cancer.

## 1 Introduction

Pancreatic cancer is one of the most common cancers and is associated with high cancer-related mortality worldwide. The incidence of pancreatic cancer has increased gradually, resulting in 331000 deaths annually [1]. Among all malignancies, pancreatic cancer has a relatively poor prognosis, with a 5-year survival rate of 5 %, which is associated with various causes, such as a high degree of malignancy, a diagnosis at an advanced stage, and a lack of effective therapy [1]. Although traditional prognostic factors such as the TNM stage, grade of histology and molecular markers are of critical prognostic value, the prognostic evaluation of patients with pancreatic cancer is still inadequate [2]. In addition, given the heterogeneity of tumors, even with similar clinical characteristics and therapies, patients with pancreatic cancer often have diverse prognoses and therapeutic effects [3]. Hence, the development of novel and valuable markers for the prognosis of patients with pancreatic cancer is urgently needed.

The tumor microenvironment (TME) is the local biological environment of solid tumors and comprises various components, such as stromal cells, endothelial cells, immune cells, and fibroblasts [4]. The TME plays an important role in all stages of cancer by affecting tumor immunity, tumor cell stemness, angiogenesis, drug resistance, migration and metastasis [5]. Hypoxia is a typical characteristic of the TME in almost all solid tumors and is due to the excessive consumption of oxygen and a shortage of oxygen supply, which results from relatively poor vascularization inside the tumor and rapid proliferation of cancer cells [6]. The abundant stromal cells are rich in the extracellular matrix, but poor vascularization causes a great degree of hypoxia within pancreatic cancer [7]. Furthermore, the hypoxic microenvironment influences the malignant phenotype, including the promotion of invasion and metastasis, which results in the development of cancer and is associated with a poor prognosis [8]. The infiltration of various immune cells, including innate and adaptive immune cells, within the cancer stroma affects the tumor immune microenvironment that varies across different types and stages of cancer [9]. Generally, a high immune infiltration status is associated with a better prognosis of the majority of solid tumors [10]. In fact, targeting the immune microenvironment has become the most promising therapy for cancer in recent years and has attracted the attention of numerous studies worldwide. The close association between hypoxia and immunity in cancer has been proven in the past. Hypoxia plays an inhibitory role in immune infiltration in the tumor microenvironment and promotes resistance to cancer immunotherapy [11]. However, the intricate mechanism still needs further investigation. Recently, with the development of whole-genome sequencing, the identification of prognostic signatures on the basis of gene expression levels has attracted increasing interest.

In the present study, a hypoxia- and immune-associated prognostic signature, which included 6 genes (*GALR2*, *AGT*, *MAPT*, *PRKCG*, *CAMK2B*, and *PKP1*), was established via LASSO Cox regression in patients with pancreatic cancer from the TCGA dataset and was further validated in 2 independent GEO datasets. Furthermore, survival analysis demonstrated that the prognostic signature was highly valuable for patients with pancreatic cancer. Finally, a nomogram was constructed with the prognostic signature and clinical characteristics, including sex, age, and histological grade.

## 2 Results

### 2.1 Evaluation of the hypoxia status and identification of related DEGs in pancreatic cancer

**Fig. 1:**
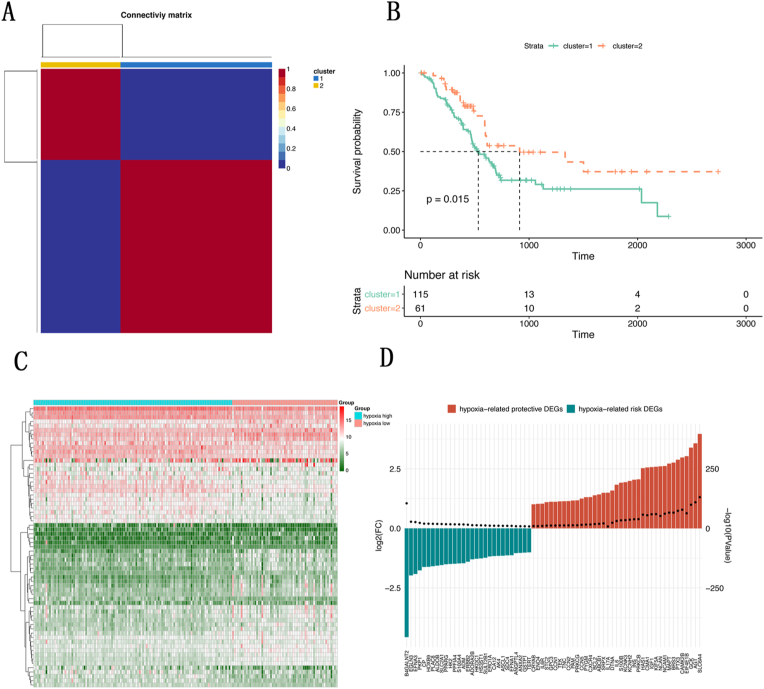
Evaluation of the hypoxia status and identification of related DEGs in pancreatic cancer. (**a**) Two hypoxia-related patient clusters were identified via the NMF method; (**b**) Survival analysis of patients in the 2 hypoxia-related clusters; (**c**) Hypoxia-related patient DEGs were identified and are displayed in a heatmap; (**d**) The expression of hypoxia-related risk and protective DEGs is displayed in a histogram.

To determine the hypoxia status in patients with pancreatic cancer, a cohort of 176 patients with pancreatic cancer from the TCGA database was used. NMF cluster analysis was applied to divide patients into 2 distinct clusters of 115 and 61 patients on the basis of the expression levels of 362 hallmark genes of hypoxia (Fig. 1a). Furthermore, the overall survival rates between the 2 clusters were compared via the Kaplan–Meier method and the log-rank test. As shown in Fig. 1b, patients in Cluster 1 had a lower overall survival rate than patients in Cluster 2 did. The results indicated that patients in Cluster 1 and Cluster 2 might have had high and low levels of hypoxia, respectively. Therefore, Cluster 1 and Cluster 2 were defined as the hypoxia^high^ and hypoxia^low^ groups, respectively. To identify hypoxia-related DEGs, differential expression analysis was performed based on the expression profiles of 362 hallmark genes of hypoxia between the hypoxia^high^ and hypoxia^low^ groups. As shown in Fig. 1c and d, a total of 68 hypoxia-related DEGs were obtained, which are displayed in a heatmap and histogram. Among them, 29 DEGs were recognized as hypoxia-related risk DEGs, with high expression in the hypoxia^high^ group, and 39 DEGs were recognized as hypoxia-related protective DEGs, with high expression in the hypoxia^low^ group.

### 2.2 Evaluation of the immune status and identification of related DEGs in pancreatic cancer

**Fig. 2:**
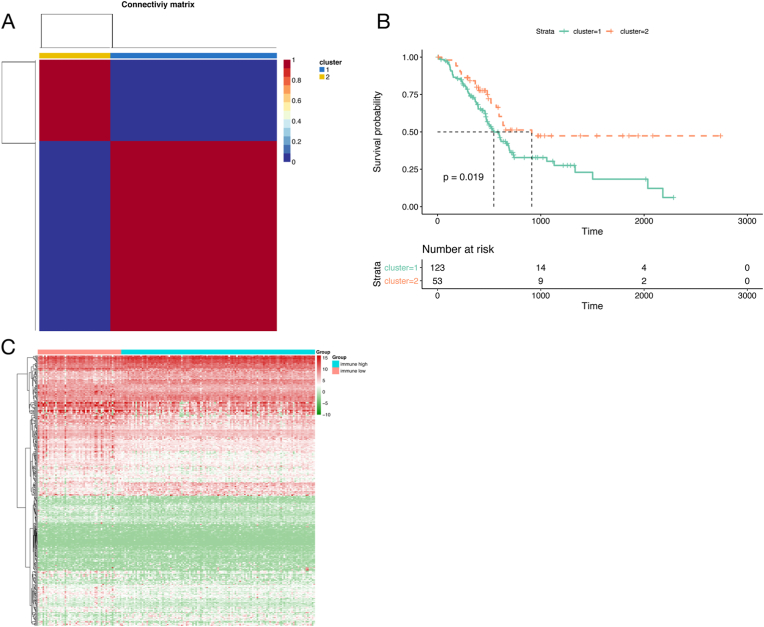
Evaluation of the immune status and identification of related DEGs in pancreatic cancer. (**a**) Two immune-related patient clusters were identified via the NMF method; (**b**) Survival analysis of patients in the 2 immune-related clusters; (**c**) Immune-related DEGs were identified and are displayed in a heatmap.

To deduce the immune status of patients with pancreatic cancer, NMF cluster analysis was used to determine the expression levels of 2166 immune hallmark genes in the discovery cohort (Fig. 2a). Two clusters were obtained that included 12,353 patients. Furthermore, a significant difference in the overall survival rates was found between these 2 clusters. As shown in Fig. 2b, patients in Cluster 1 had poorer prognosis outcomes than patients in Cluster 2 did. These results indicated that patients in Cluster 1 and Cluster 2 might have had low and high immune statuses, respectively. Accordingly, Cluster 1 and Cluster 2 were considered the immune^high^ and immune^low^ groups, respectively. To identify immune-related DEGs, differential expression profiling of the 2166 immune hallmark genes between the immune^high^ and immune^low^ groups was performed. As shown in Fig. 2c, a total of 343 immune-related DEGs were identified, which are displayed in a heatmap. Among them, 235 DEGs were regarded as immune-related protective DEGs, with high expression in the immune^high^ group, and 108 DEGs were regarded as immune-related risk DEGs, with high expression in the immune^low^ group.

### 2.3 Identification of hypoxia- and immune-related DEGs in pancreatic cancer

**Fig. 3:**
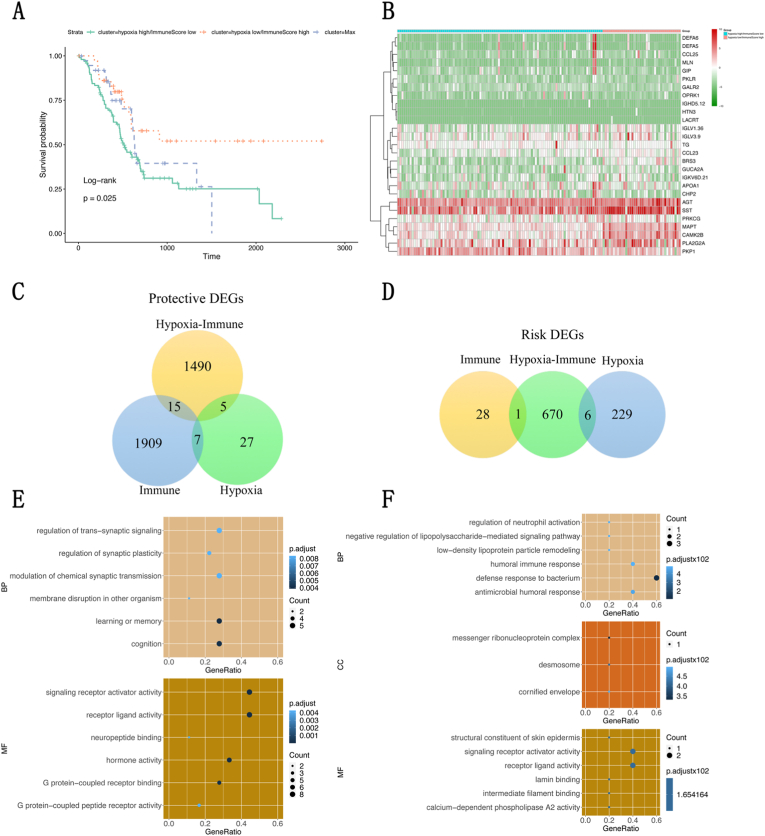
Identification of hypoxia- and immune-related DEGs in pancreatic cancer. (**a**) Survival analysis among the hypoxia^low^-immune^high^, hypoxia^high^-immune^low^, and mixed groups; (**b**) Hypoxia- and immune-related DEGs are displayed in a heatmap; (**c**, **d**) A Venn diagram is used to display the overlaps among hypoxia- and immune-related DEGs, hypoxia-related DEGs and immune-related DEGs; (**e**, **f**) Results of Gene Ontology enrichment analyses of protective and risk-related DEGs.

Based on the status of hypoxia and immunity, as determined above, all patients were assigned to 3 groups, namely, the hypoxia^low^-immune^high^, hypoxia^high^-immune^low^, and mixed groups. The overall survival rates in the 3 groups were analyzed via the Kaplan–Meier method and log-rank test. As shown in Fig. 3a, there were significant differences in overall survival among the 3 groups, with the best overall survival in the hypoxia^low^-immune^high^ group and the poorest overall survival in the hypoxia^high^-immune^low^ group. To identify hypoxia- and immune-related DEGs, differential expression analysis was performed by using the expression profiles of all the genes from the TCGA database between the hypoxia^low^-immune^high^ and hypoxia^high^-immune^low^ groups. As shown in Fig. 3b, a total of 2187 hypoxia- and immune-related DEGs were obtained, which are displayed in a heatmap. Among them, 1510 DEGs were regarded as hypoxia- and immune-related protective DEGs, with high expression in the hypoxia^low^-immune^high^ group, and 677 DEGs were regarded as hypoxia- and immune-related risk DEGs, with high expression in the hypoxia^high^-immune^low^ group.

Furthermore, we further analyzed the DEGs by overlapping those related to hypoxia, immunity and hypoxia and immunity. As shown in Fig. 3c and d, a total of 27 critical DEGs were identified and are displayed in a Venn diagram, including 20 protective DEGs and 7 risk-related DEGs. Among all the protective DEGs, the majority (15 out of 20) were immune-related DEGs. Among all the risk-associated DEGs, the majority (6 out of 7) were hypoxia-related DEGs. These results suggested that a high immune status might be beneficial for patients with pancreatic cancer. In contrast, a high level of hypoxia was related to a poor prognosis in patients with pancreatic cancer. Moreover, Gene Ontology enrichment analyses were performed for the protective and risk-related DEGs. The results are shown in Fig. 3e and f.

### 2.4 Construction of a prognostic signature with hypoxia- and immune-related DEGs

**Fig. 4:**
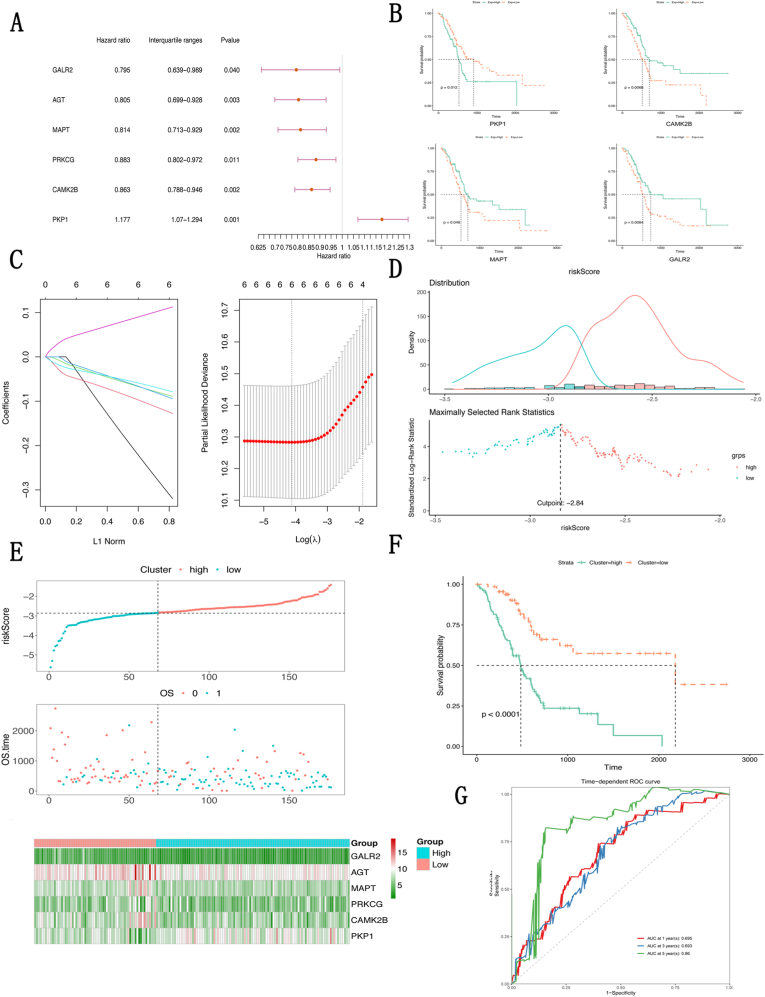
Construction of a prognostic signature with hypoxia- and immune-related DEGs. (**a**) Forest plot of the 6 prognostic DEGs selected by univariate Cox regression; (**b**) There was a significant difference in the overall survival rates between the groups with high and low expression of 4 DEGs (*GALR2*, *MAPT*, *CAMK2B*, and *PKP1*); (**c**) Six prognostic DEGs were identified via LASSO regression; (**d**) The optimal cutoff for risk scores was determined by maximally selected rank statistics; (**e**) Distributions of the risk scores and overall survival times and a heatmap of the expression of the 6 prognostic DEGs in all patients with pancreatic cancer; (**f**) Survival analysis of patients between the high- and low-risk groups; (**g**) Time-dependent ROC curve of the prognostic signature.

To identify hypoxia- and immune-related prognostic DEGs in patients with pancreatic cancer, univariate Cox regression was performed with the 27 DEGs identified above. In Fig. 4a, 6 DEGs (*GALR2*, *AGT*, *MAPT*, *PRKCG*, *CAMK2B*, and *PKP1*) with prognostic value are displayed in a forest plot. Furthermore, overall survival was compared between the 2 groups according to the median expression levels of the prognostic DEGs. As shown in Fig. 4b, patients with high expression of *GALR2*, *MAPT*, and *CAMK2B* had better survival than those with low expression did. Conversely, patients with low expression of *PKP1* had better survival than those with low expression did.

To construct a prognostic signature, LASSO regression was further utilized to identify the most valuable DEGs. The results revealed that all the 6 DEGs obtained via univariate Cox regression had important prognostic value, and therefore, they were used for the construction of a prognostic signature to calculate the risk score for each patient with pancreatic cancer (Fig. 4c). To evaluate the prediction efficacy of the prognostic signature constructed, the patients with pancreatic cancer were divided into 2 groups according to the optimal cutoff for risk scores, which was determined with maximally selected rank statistics (Fig. 4d). The risk score and overall survival time of each patient and a heatmap of all the prognostic DEGs are shown in Fig. 4e. Survival analysis revealed that patients in the low-risk group had better overall survival than did those in the high-risk group (Fig. 4f). In addition, a time-dependent ROC curve revealed the excellent prediction ability of the prognostic signature, with an AUC of 0.713 (Fig. 4g).

### 2.5 Validation of the prognostic signature

**Fig. 5:**
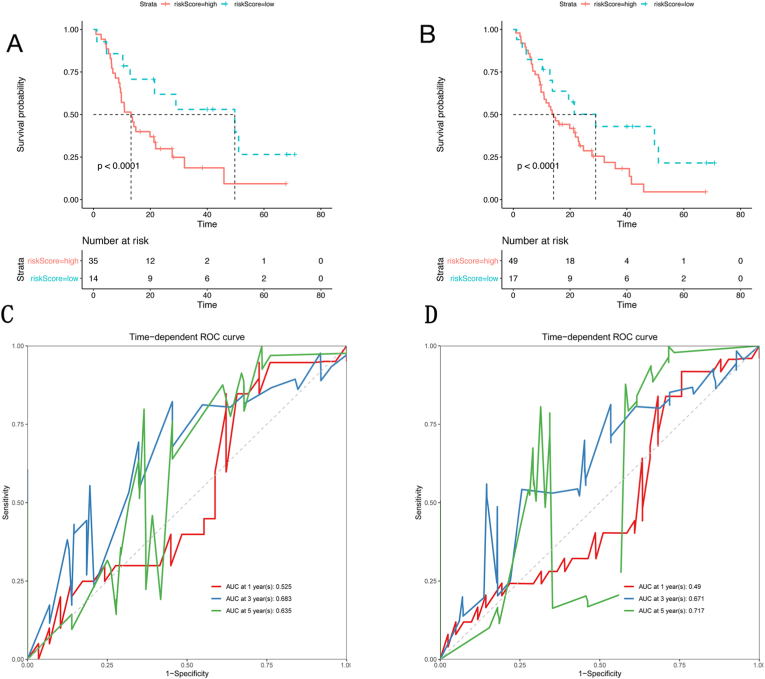
Validation of the prognostic signature. (**a**, **b**) Survival analysis of patients at high and low risk and time-dependent ROC curve analysis of the prognostic signature were performed in the GSE78229 and GSE62452 cohorts.

To evaluate the applicability of the prognostic signature, 2 cohorts (GSE78229 and GSE62452) from the GEO database were employed for validation. The risk scores were calculated via the abovementioned formula, and the patients from the 2 cohorts were divided into 2 groups via the method of maximally selected rank statistics. As shown in Fig. 5a–d, a significantly higher overall survival rate was observed in the low-risk group than in the high-risk group in the 2 cohorts. However, the time-dependent ROC curve showed an unsatisfactory AUC value, indicating the suboptimal accuracy of the risk score model in the validation cohort.

### 2.6 Performance of the prognostic signature in subgroups of patients based on clinical factors of pancreatic cancer

**Fig. 6:**
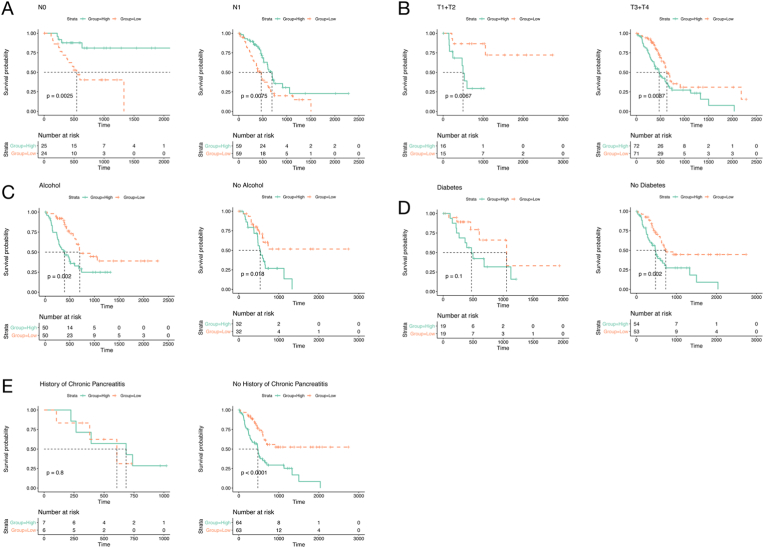
Validation of the prognostic signature in subgroups according to various clinical factors. (**a**–**e**) Survival analysis of patients at high and low risk was performed in subgroups according to various clinical factors, including the N stage, pathological T stage, alcohol history, history of diabetes, and history of chronic pancreatitis.

To test the applicability of the prognostic signature in subgroups of patients based on various clinical factors, stratification analysis of overall survival was performed with various clinical factors, including the pathological N stage, pathological T stage, alcohol history, history of diabetes, and history of chronic pancreatitis. The patients in each subgroup were further divided into 2 groups according to the median risk score, and survival analysis was performed. As shown in Fig. 6a–e, patients at high risk in all the subgroups based on the pathological T stage and alcohol history, as well as in the subgroup with no history of chronic pancreatitis or diabetes. exhibited significantly poorer survival than did patients at low risk. Conversely, patients at low risk had significant survival benefits compared with those at high risk in both subgroups of the pathological N stage. However, there was no significant difference in survival between patients at high and low risk in the subgroup with a history of diabetes or chronic pancreatitis.

### 2.7 Analysis of immune cell infiltration in pancreatic cancer

**Fig. 7:**
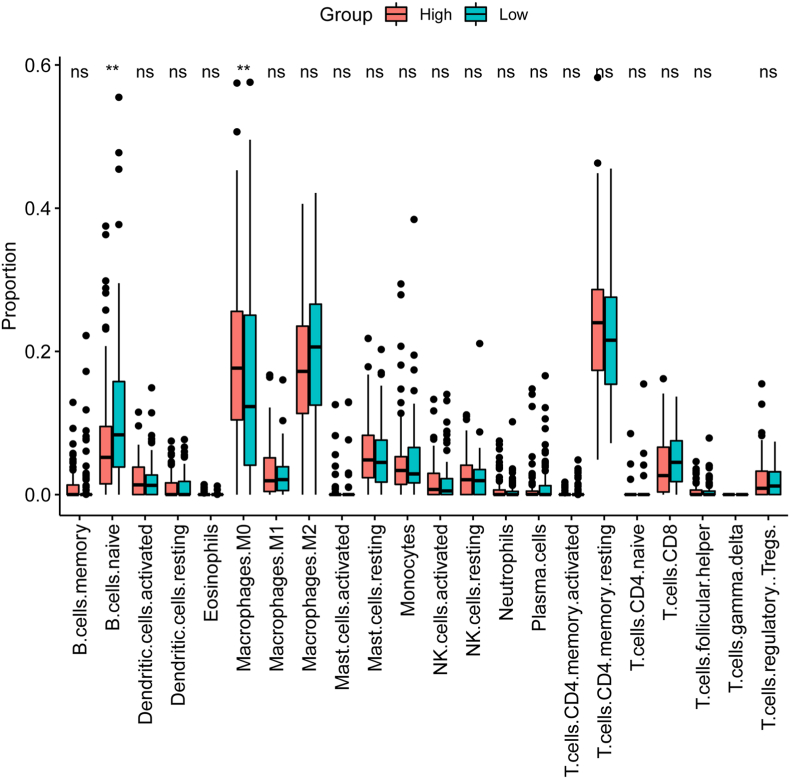
Analysis of the relationship between the prognostic signature and immune cell infiltration in pancreatic cancer.

Given the difference in the immune status between patients with pancreatic cancer, the infiltration of a total of 22 immune cell subtypes was analyzed with CIBERSORT between patients at high and low risk. As shown in Fig. 7, significant differences in the infiltration of naïve B cells and M0 macrophages were detected between the high- and low-risk score groups.

### 2.8 Construction of a nomogram

**Fig. 8:**
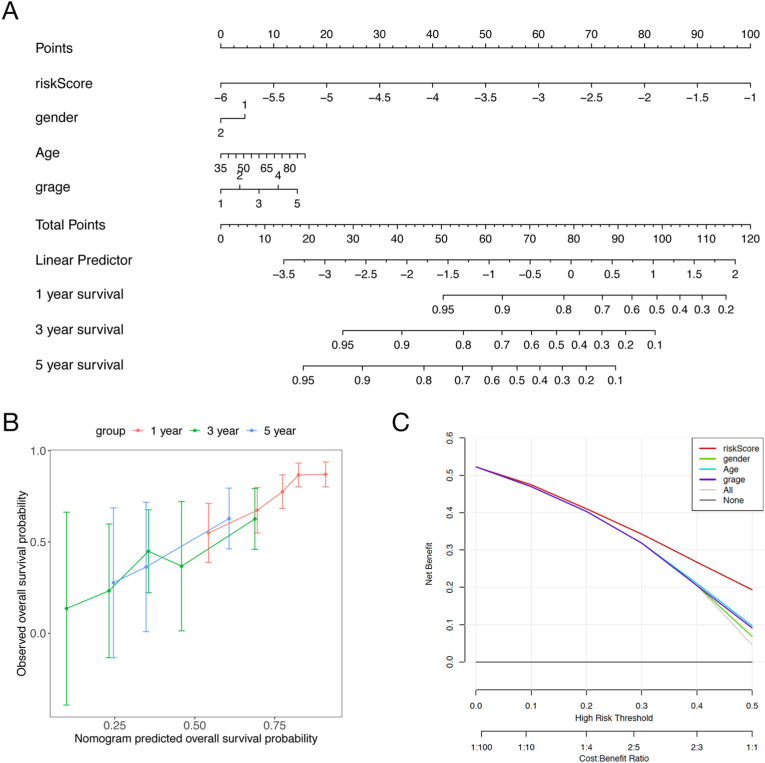
Construction of the nomogram. (**a**) A nomogram was constructed with the prognostic signature and various prognosis-related clinical factors, including sex, age and histological grade; (**b**) The calibration curves for 1-, 3-, and 5-year survival were used to evaluate the performance of the nomogram; (**c**) Decision curve analysis (DCA) was performed to evaluate the clinical applicability of the prognostic signature.

Nomograms are important visualization tools for survival prediction on the basis of various prognostic factors for individuals. Herein, we constructed a nomogram for the prediction of 1-, 3-, and 5-year survival in the discovery cohort, with the prognostic signature and various prognosis-related clinical factors, including sex, age and histological grade. As shown in Fig. 8a, the prognostic signature, sex, age and histological grade were mapped to points according to their corresponding contributions to overall survival, and the points were summed to obtain a total point for survival prediction. Furthermore, calibration curves for 1-, 3-, and 5-year survival were applied to evaluate the performance of this nomogram. As shown in Fig. 8b, the calibration curves demonstrated the excellent ability of this nomogram to predict survival at 1, 3, and 5 years. In addition, decision curve analysis (DCA) indicated that the prognostic signature was more clinically useful than sex, age and histological grade (Fig. 8c).

## 3 Discussion

High malignancy and unsatisfactory therapy lead to a poor prognosis of patients with pancreatic cancer, even though many studies have focused on this disease. Given the tumor heterogeneity, currently used factors are insufficient for evaluating the prognosis and for clinical decision making for patients with pancreatic cancer [3]. Exploitation of reliable prognostic markers for accurate prediction of individual survival is highly valuable for the personalized treatment of patients with pancreatic cancer. With the rapid development of high-throughput sequencing over the past decade, the identification of prognostic signatures related to the expression of specific genes has attracted much interest for patients with pancreatic cancer.

Hypoxia and immunity are crucial characteristics of the TME and are closely associated with cancer prognosis [11]. Biomarkers of the tumor immune microenvironment are highly valuable in detecting cancer and evaluating patient prognosis and treatment effects as supplements to the classical clinical characteristics of cancer [12]. Hypoxia is a TME phenomenon resulting from excessive proliferation of cancer cells and relative oxygen deficiency within the tumor. Alternatively, enhanced anaerobic glycolysis is responsible for the energy supply within the hypoxic region of cancer [6]. The hypoxia status is an independent prognostic factor for cancer and is related to the suppression of immune cell infiltration [11]. Prognostic signatures established with multiple genes related to a variety of tumor phenotypes, such as a 6-gene signature related to m^6^A regulators, an 8-gene hypoxia-related signature and a 15-gene signature related to immune and stromal cell proliferation, have been reported in several studies [[13], [14], [15]]. NMF is a robust method that is frequently used in the classification of expression profiles according to specific features and has been applied to explore subtypes related to the immunity of lung cancer [16]. In the present study, NMF was used to classify patients with pancreatic cancer into 2 clusters based on the expression levels of hallmark genes of hypoxia and immunity. Through differential expression analysis, 27 critical hypoxia- and immune-related DEGs were identified. Interestingly, most protective DEGs were related to immunity, whereas most risk-associated DEGs were related to hypoxia. These results indicate that immunity is beneficial, but hypoxia has adverse effects on pancreatic cancer, which is consistent with previous reports [6,10]. Furthermore, a 6-gene hypoxia- and immune-related prognostic signature was constructed for pancreatic cancer via LASSO regression. A time-dependent ROC curve indicated the excellent performance of the prognostic signature, with an AUC of 0.713. Moreover, the prognostic signature exhibited a stable performance in 2 validation cohorts from the GEO database. Through stratification analysis, the efficacy of the prognostic signature was verified for all the subtypes of various crucial clinical factors, except for subtypes of patients with diabetes and chronic pancreatitis. This might be due to the limited number of patients with diabetes and chronic pancreatitis. Immune infiltration analysis demonstrated that naïve B cells and M0 macrophages were associated with the prognostic signature, suggesting significant roles for naïve B cells and macrophages in the tumor microenvironment of pancreatic cancer. These results are consistent with those of a previous study that revealed the role of M0 macrophages in pancreatic ductal adenocarcinoma [17]. In addition, a nomogram was established with the prognostic signature and clinical factors such as sex, age, and histological grade. Taken together, the prognostic signature and the nomogram might be promising supplements to previous prognostic evaluation indicators for pancreatic cancer.

Among the prognostic signature genes, *GALR2*, *AGT*, *MAPT*, *PRKCG*, and *CAMK2B* were found to be protective DEGs, whereas *PKP1* was a risk-related DEG. *GALR2*, a receptor for galanin, is expressed in many normal tissues and several cancers [18,19]. Several studies have reported that the role of GALR2 in various cancers involves angiogenesis promotion, inhibition of cancer proliferation, induction of apoptosis and facilitation of perineural invasion [[20], [21], [22]]. Angiotensinogen (*AGT*), the precursor of the angiotensin peptide, is traditionally regarded as a regulator of the renin–angiotensin system [23]. However, accumulating evidence has shown that the function of *AGT* is closely associated with cancer. The upregulated expression and hypomethylation of *AGT* were found in gastric cancer, and there was a negative association between the prognosis of gastric cancer and *AGT* expression [24]. Suppression of *AGT* by high-glucose treatment plays a critical role in the promotion of proliferation and metastasis in breast cancer, suggesting that *AGT* is a cancer suppressor gene [25]. The microtubule-associated protein tau (*MAPT*) gene is responsible for encoding the tau protein and is associated mainly with various neurodegenerative disorders [26]. Recently, the function of *MAPT* in cancer has been investigated. In view of its prognostic value, *MAPT* has been included in immune-related prognostic signatures for patients with hepatocellular carcinoma and esophageal carcinoma [27,28]. Moreover, downregulation of *MAPT* was observed in 786-O clear cell renal cell carcinoma cells under long-term hypoxia [29]. In pancreatic cancer, *MAPT* has been recognized as a predictive marker of the treatment effect of sulfur benzoylphenylurea analogs (SG410 and SG430), and the methylation of *MAPT* in plasma has been detected for the noninvasive diagnosis of pancreatic cancer [30,31]. *PRKCG*, one of the isozymes of protein kinase C (*PKC*), has been associated mainly with diseases of the nervous system in previous studies. However, increasing evidence indicates that *PRKCG* may play essential roles in cancer and immunity. Retroviral transduction of *PRKCG* into murine cytotoxic T lymphocytes demonstrated that *PRKCG* might be involved in the signaling pathway of proliferation activation in T cells [32]. *C1B5*, a synthetic subdomain peptide of *PRKCG*, has shown anticancer potential in pancreatic cancer via the activation of T and NK cells in a study investigating the effect of cotreatment with *C1B5* and gemcitabine in a pancreatic cancer mouse model [33]. In addition, a previous study revealed that *PRKCG* was upregulated in colon cancer and promoted the migration of colon cancer cells [34]. Several studies have investigated the physiological and pathological functions of calmodulin-dependent protein kinase 2b (*CAMK2B*) in different tissues [35]. For example, *CAMK2B* prevented the induction of apoptosis by homocysteine in neurons through the activation of the *HIF-1* signaling pathway, which mainly mediates the signal transduction of hypoxia in cells [36]. However, only a few studies have focused on the role of *CAMK2B* in cancer. Hypermethylation of *CAMK2B* has been reported in breast cancer compared with normal breast tissue, indicating that promoter methylation of *CAMK2B* is an epigenetic marker for breast cancer [37]. The pathological role of plakophilin (*PKP1*) is associated mainly with dysplasia and cancer [[38], [39], [40]]. *PKP1* expression is inversely correlated with the histological grade, recurrence and metastasis in patients with oropharyngeal cancer [40]. Loss of *PKP1* promoter methylation could lead to the transition of Barrett's esophagus to esophageal cancer through a decrease in desmosome assembly and an increase in cell motility [39].

To the best of our knowledge, no study has reported a prognostic signature related to hypoxia and immunity in pancreatic cancer. In the present study, we established a 6-gene hypoxia- and immune-related prognostic signature that was an independent prognostic factor for pancreatic cancer. However, there are several limitations to our study. First, the TCGA datasets that were used to construct the signature did not include enough samples. Therefore, additional cohorts from different databases or collect data from multi-institutional studies that includes more samples are still needed to improve this prognostic signature. Second, the TCGA dataset cohort consisted of patients with more than one ethnicity. The efficacy of the application of this prognostic signature in particular populations still needs validation in independent cohorts with a single race. Finally, the function and expression of these 6 genes in pancreatic cancer need to be confirmed via in vivo and in vitro experimentsin in the future. Taken together, the results of the present study provide a promising prognostic signature that may be helpful in practical applications because of the reduced necessity for whole-genome sequencing in patients with pancreatic cancer. In addition, more evidence is needed for the clinical applicability of this signature in patients with pancreatic cancer in future studies.

## 4 Materials and methods

### 4.1 Data collection

A discovery cohort of 176 samples from patients with pancreatic cancer was obtained from The Cancer Genome Atlas (TCGA) database (https://portal.gdc.cancer.gov/). Level 3 RNA-seq transcriptome data and corresponding clinical information of patients with pancreatic cancer were used for subsequent analysis. External validation was performed with two independent cohorts of patients with pancreatic cancer, namely, the GSE78229 and GSE62452 datasets, from the Gene Expression Omnibus (GEO) database (https://www.ncbi.nlm.nih.gov/geo/). All the data from the TCGA and GEO databases conformed to the database policy for data acquisition and analysis, with no need for informed consent and approval from the institutional review board.

### 4.2 Acquisition of hallmark genes of hypoxia and immunity

A total of 362 hallmark genes of hypoxia were obtained through the integration of genes from 3 databases, including 200 genes from the MSigDB (http://www.zhounan.org/ferrdb/operations/download.html), 104 genes from the NCBI (https://www.ncbi.nlm.nih.gov/gene/?term=(hallmark+hypoxia)+AND+%22Homo+sapiens%22%5Bporgn%3A__txid9606%5D), and 110 genes from the KEGG HIF-1 signaling pathway (https://www.genome.jp/entry/map04066). To obtain hallmark genes associated with immunity, the ImmPort database (https://www.immport.org/shared/genelist) and the Innate database (https://www.innatedb.com/annotatedGenes.do?type=innatedb) were utilized. In total, 2166 hallmark genes of immunity were obtained through the integration of 1509 genes from the ImmPort database and 1052 genes from the Innate database.

### 4.3 Evaluation of the hypoxia status and identification of hypoxia-related DEGs

To determine the hypoxia status of patients with pancreatic cancer, nonnegative matrix factorization (NMF) was utilized to classify all patients in the discovery cohort into two clusters based on the expression levels of hallmark genes of hypoxia. The hypoxia^high^ and hypoxia^low^ groups were subsequently identified on the basis of these two clusters. Furthermore, differentially expressed genes (DEGs) among 362 hallmark genes of hypoxia were identified between the hypoxia^high^ and hypoxia^low^ groups via the edgeR package in R, with the criteria of an FDR<0.05 and |log2(FC)|>1.

### 4.4 Evaluation of the immune status and identification of immune-related DEGs

The immune status was determined via the same method that was used to determine the hypoxia status. Two groups, namely, the immune^high^ and immune^low^ groups, were ultimately identified. In addition, DEGs among 2166 immune hallmark genes were identified between the immune^high^ and immune^low^ groups via the edgeR package in R.

### 4.5 Identification of hypoxia- and immune-related prognostic DEGs

Based on the status of hypoxia and immunity, as determined above, all the patients were further divided into 3 groups, namely, the hypoxia^high^-immune^low^ group, the hypoxia^low^-immune^high^ group, and the mixed group. Furthermore, hypoxia- and immune-related DEGs were identified between the hypoxia^high^-immune^low^ and hypoxia^low^-immune^high^ groups via the edgeR package in R. Through the overlap of all the DEGs obtained, protective and risk-related DEGs were identified. The protective DEGs were identified based on high expression in the hypoxia^low^, immune^high^ and hypoxia^low^-immune^high^ groups. The risk-related DEGs were identified based on high expression in the hypoxia^high^, immune^low^ and hypoxia^high^-immune^low^ groups. To identify hypoxia- and immune-related prognostic DEGs, univariate Cox regression analysis was performed with all the protective and risk-related DEGs at a significance level of p < 0.05. Furthermore, the patients were divided into high- and low-expression groups according to the median gene expression level, and survival analysis was used to compare overall survival between the groups with high and low expression of prognostic DEGs.

### 4.6 Construction of the prognostic gene signature

Least absolute shrinkage and selection operator (LASSO) has been broadly employed to reduce high-dimensional data to screen for prognosis-related factors. In this study, LASSO was applied to further select the critical prognostic DEGs from those obtained based on the results of univariate Cox regression analysis. A prognostic signature was subsequently constructed to obtain the risk score for each patient via the following formula: risk score = Σ (coefficient_i_ × expression_i_ of prognostic gene_i_). Furthermore, the optimum cutoff for risk scores was determined via the method of maximally selected rank statistics in R and was used to distinguish patient groups with high- and low-risk scores.

### 4.7 Nomogram construction

A nomogram is a method of visualizing survival prediction through the incorporation of variables selected by multivariate Cox regression as a single numerical value. In this study, to better predict the overall survival of patients with pancreatic cancer at 1, 3, and 5 years, a nomogram was constructed with the prognostic signature that we identified and various clinical characteristics. Calibration plots of 1-, 3-, and 5-year survival and decision curve analysis (DCA) were further utilized to assess the nomogram performance.

### 4.8 Analysis of immune cell infiltration

To explore the relationship between the prognostic signature and immune cell infiltration in patients with pancreatic cancer, CIBERSORT was used to analyze the differences in the infiltration of 22 immune cell subtypes (LM22) between patients with high- and low-risk scores. The permutation was set at 100.

### 4.9 Statistical analysis

All the statistical analyses were performed with the R software (version 4.1.0), and NMF was performed with the “NMF” package. The “edgeR” package was used to screen for DEGs, with the criteria of an FDR<0.05 and |log2(FC)|>1. The “pheatmap” package was used to generate a heatmap. Gene Ontology enrichment analysis was performed with the “clusterProfiler” package. The “survminer” package was used to perform Cox proportional hazard regression analysis. The “glmnet” package was used to perform LASSO regression. The “rms” package was used to plot the nomogram and a calibration plot. Maximally selected rank statistics were performed with the “maxstat” package in R. The Kaplan–Meier method and log-rank test were used for survival analysis with the “survival” package in R. The “forestplot” package was used for forest plots. The CIBERSORT package was used to perform immune infiltration analysis. A time-dependent receiver operating characteristic (ROC) curve was plotted with the “survival” package in R. The “VennDiagram” package was used to plot a Venn diagram. P < 0.05 was considered statistically significant.

## Author contributions

Wang Zheng and Xu Qinhong designed and supervised the study. Yang Ganghua, Yu Jiawei and Lu Zhengyang were responsible for the online data search, acquisition and interpretation. Meng Fandi and Wan Yong were responsible for data extraction and analysis. Yu Jiawei, Li Xuqi, and Yang Ganghua were responsible for writing the manuscript. All the authors read and approved the final manuscript.

## Institutional review board statement

Not applicable. All data used in this study are publicly available.

## Disclaimer/publisher's note

The statements, opinions and data contained in all publications are solely those of the individual author(s) and contributor(s) and not of MDPI and/or the editor(s). MDPI and/or the editor(s) disclaim responsibility for any injury to people or property resulting from any ideas, methods, instructions or products referred to in the content.

## Funding

This study was supported by the Scientific and Technological Development Research Project Foundation of Shaanxi Province (No. 2023-YBSF-309 and No. 2020JQ-513).

## Declaration of competing interest

All the authors declare that there are no financial and other conflicts of interest in relation to this study.

## Data Availability

All the data obtained in this study can be found in the TCGA (https://portal.gdc.cancer.gov/) and Gene Expression Omnibus (GEO) (https://www.ncbi.nlm.nih.gov/geo/) repositories.
